# Evaluation of ivc and ijv dimensions in prediction of fluid responsiveness in spontaneous breathing patients with septic shock

**DOI:** 10.1186/2197-425X-3-S1-A594

**Published:** 2015-10-01

**Authors:** A Hasanin, A Lotfy, IR Abdel-Aal, J Elkholey, A El-Adawy, H Mohamed, A Zaghlol, M Salem, A Mukhtar

**Affiliations:** Cairo University, Anesthesia and Critical Care Medicine, Cairo, Egypt

## Introduction

Prediction of fluid responsiveness (FR) is a critical step in management of patients with septic shock. Using ultrasound in detection of inferior vena cava (IVC) diameters and collapsibility is established in mechanically ventilated patients; however its use in spontaneous breathing patients is still controversial [1]. Few studies reported a correlation between internal jugular vein dimensions and central venous pressure (CVP) [2, 3] but no data are available about the use of IJV dimensions in detection of FR.

## Objectives

The aim of our study is to determine the possible rule of IVC diameters, collapsibility, and IJV dimensions in prediction of FR in spontaneous breathing patients.

## Methods

Twenty spontaneous breathing patients with septic shock were included in the study. Ultrasound examination was done before fluid resuscitation. IVC minimal and maximum diameters, IVC collapsibility index (IVC maximum - IVC minimum/IVC maximum), IJV cross sectional area, IJV/Common carotid artery (CCA) ratio, and IJV aspect ratio (IJV vertical diameter/IJV transverse diameter) were measured before fluid resuscitation. Transthoracic echocardiography (TTE) was done to determine FR which was defined as increase in sub-aortic velocity time integral (VTI) > 15% after fluid bolus 7 ml/Kg. Sensitivity, Specificity and Area under receiver operating characteristic (AUROC) curves were determined for all ultrasound parameters as well as CVP for detection of FR.

## Results

Eleven patients showed FR and nine patients were non-responders. APACHE II score was 16 ± 6.3, age was 36 ± 15 years. AUROC curve (95% CI) for prediction of FR was: 0.51(0.27-0.73) for **CVP** with sensitivity 27% and specificity 100% at cutoff value 9 mmHg, 0.5(0.27-0.72) for **IJV aspect ratio**, 0.56(0.33-0.78) for **IJV/CCA**, 0.54(0.31-0.77) for **IJV surface area**, 0.52(0.28-0.74) for **IVC collapsibility index**, 0.72(0.53-0.92) for **IVC minimum diameter** with sensitivity 45% and specificity 89% at cutoff value 0.3 cm, 0.76(0.48-0.90) for **IVC maximum diameter** with sensitivity 82% and specificity 67% at cutoff value 1.5 cm.

## Conclusions

Our finding supports the evidence of the poor value of most of static parameters in prediction of FR in spontaneous breathing patients. IVC minimum and maximum diameters were the best parameters in detection of FR in our cohort.
